# The Healthy Brain Initiative—expanding public health capacity to address dementia

**DOI:** 10.1093/geront/gnaf220

**Published:** 2025-10-07

**Authors:** Meghan Fadel, John Shean, Eva Jackson, Janicka D Harris, Juan Rodríguez, Shelby Sutton Roberts

**Affiliations:** Division of Programs, Alzheimer’s Association, Chicago, Illinois, United States; Division of Programs, Alzheimer’s Association, Chicago, Illinois, United States; Division of Programs, Alzheimer’s Association, Chicago, Illinois, United States; Division of Programs, Alzheimer’s Association, Chicago, Illinois, United States; Division of Programs, Alzheimer’s Association, Chicago, Illinois, United States

**Keywords:** Dementia, Brain health, Public policy, Social determinants of health

## Abstract

In 2005, congressional support led to the creation of the Healthy Brain Initiative (HBI) and the collaboration between the Alzheimer’s Association and the Centers for Disease Control and Prevention to prioritize brain health in public health practice. Over 20 years, the HBI has developed and implemented the HBI Road Map Series to increase the capacity of health departments to integrate dementia into health departments nationwide, aligning frameworks like the Essential Public Health Services and focusing on health equity across the life course. A growing number of HBI partners now work together to implement public health strategies that promote brain health, address dementia, and support people living with dementia and caregivers. Recognizing opportunities to influence the trajectory of public health action, the HBI prioritizes growing the availability and use of dementia-related public health data and equipping the public health workforce with the knowledge and confidence to make change. This article documents the history and evolution of the HBI, including a description of current efforts and the broader public health context to which it has contributed; efforts of the HBI and partners in national, state, local, territorial, and tribal public health agencies have led to transformative change.

Alzheimer’s disease is a significant public health issue requiring a robust response. With over 7 million Americans living with Alzheimer’s and the prevalence projected to increase to nearly 13 million by 2050, population health interventions are essential to support risk reduction; increase access to early detection, diagnosis and treatment; and improve the lives of people living with dementia, their caregivers, and their communities (­Alzheimer’s Association, 2025a).

Over the last 20 years, advancements in the science and practice of dementia risk reduction, early detection of dementia, and care and support of people living with dementia and their caregivers have changed how public health agencies respond to and address this growing issue. Population-level interventions can mitigate risk in communities and enhance systems of care, improving outcomes for people with dementia and their caregivers (Alzheimer’s Association and Centers for Disease Control and Prevention, 2018). As evidenced with other chronic diseases–including heart disease, diabetes, and cancer–public health approaches can reduce the burden of disease and reduce health disparities related to dementia (Jackson et al., 2023; Jones et al., 2018; Weiss & Shean, 2021).

In 2005, Congress recognized the potential for public health impact in the prevention, treatment, and management of ­Alzheimer’s disease by providing the first appropriations to the Centers for Disease Control and Prevention (CDC). This funding led to the development of the Healthy Brain Initiative (HBI), a public-private partnership mobilizing the nationwide public health response to dementia. Today, the HBI illustrates the longstanding cooperative agreement between the CDC and the Alzheimer’s Association, along with a growing collaborative of national partners, seeking to advance understanding of and support for brain health as a central component of public health practice. In the two decades since its inception, the HBI has supported the public health field to address Alzheimer’s across the life course, providing a framework for national, state, local, territorial, and tribal public health jurisdictions to accelerate progress (Weiss & Shean, 2021).

The HBI strategically aligns with well-recognized public health concepts, including the Essential Public Health Services (EPHS) framework, which identifies public health activities for all communities to improve population health outcomes (CDC, 2024a). This framework informs the focus of health departments nationally and the framework of the HBI. HBI actions across the three levels of public health prevention (primary, secondary, and tertiary) are necessary to ensure all communities benefit from population health interventions (Alzheimer’s Association and Centers for Disease Control and Prevention, 2023). The HBI directs public health attention to the social determinants of health (SDOH) (CDC, 2020)—including access to health care and social services, affordable and safe housing, quality education, nutritious food, physical activity opportunities, transportation, adequate household income, and safe neighborhoods–and identifies ways to positively impact SDOH that influence brain health, dementia risk, and access to treatment and services (Adkins-Jackson et al., 2023;  Coogan et al., 2020; Freudenberg et al., 2015).

## HBI: A central pillar of public health action

The continued evolution of the HBI has helped achieve many of the public health successes seen over the past 20 years. This paper will describe how the HBI has:

1. Developed, disseminated, and supported the implementation of the HBI Road Map Series in all 50 states and the District of Columbia;
2. Advanced the collection, analysis, and use of public health data, resulting in national estimates for cognitive decline and caregiving;
3. Increased knowledge among the workforce, training thousands of public health students and professionals; and
4. Contributed to measurable public health progress on policy and programs nationwide, including the implementation of several federal laws, and an increase in legislative action and funding appropriations across states.

## The HBI Road Map Series

Upon creation of the HBI, the CDC and the Alzheimer’s Association completed a public health landscape analysis, resulting in 44 strategic actions for national partners to undertake. These actions laid the foundation for the 2007 publication of the first Road Map. Since then, six Road Maps have been published in the series (Table 1). Each HBI Road Map recommends public health actions that guide state, local, territorial, and tribal agencies nationwide toward collective impact. The series is iterative, building on the successes of previous editions, ensuring the recommended actions evolve over time, reflecting advances in science, public health practice, and federal policy. Nationwide dissemination of the series has also increased with each edition, reaching a broader audience of public health professionals.

**Table 1.** Overview of the HBI Road Map Series.

Publication year	Title	Description	Dissemination (as of June 2025)
2007	*National Public Health Road Map to Maintaining Cognitive Health*	The first edition established the foundational framework for viewing cognitive health and dementia as a public health issue. It outlined 44 actions to address dementia at the national level and laid the foundation for the public health community more broadly to engage on the issue.	No data available
2013	*The Public Health Road Map for State and National Partnerships, 2013–2018*	The 2013 update expanded the role that state and local public health departments and partners could play by offering 35 actions to promote cognitive functioning, address cognitive impairment and dementia and help meet the needs of caregivers.	No data available
2018	*State and Local Public Health Partnerships to Address Dementia, The 2018–2023 Road Map*	The third edition continued to chart a course for state and local public health departments and their partners. This publication prepared communities to act quickly and strategically by stimulating changes in policies, systems, and environments. As a new feature of this edition, the alignment of 25 actions with Essential Public Health Services ensured that initiatives to address Alzheimer’s could be incorporated easily and efficiently into existing public health efforts.	2,213 hard copies 5,065 digital downloads
2019	*The Healthy Brain Initiative: Road Map for Indian Country*	This publication was the first public health guide focused on dementia in American Indian and Alaska Native communities. The guide offered eight broad public health strategies designed to support discussion about dementia and caregiving within tribal communities and to encourage a public health approach as part of a larger holistic response.	2,490 hard copies; 1,162 digital downloads
2023	*The Healthy Brain Initiative: State and Local Road Map for Public Health, 2023–2027*	The 2023 iteration advances equity by fully integrating brain health and caregiving into state and local public health practice and addresses SDOH across the life course. In this edition, the conceptual framework evolved to incorporate equity at its center, the Road Map prioritized community partnerships as foundational, and added collateral resources, including an implementation guide and evaluation tool.	10,156 hard copies; 5,694 digital downloads
2024	*The Healthy Brain Initiative: Road Map for American Indian and Alaska Native Peoples*	The second edition, focused on American Indian and Alaska Native (AI/AN) people offers expanded actions for a strength-based public health approach in communities. Guided by a Leadership Committee of tribal leaders, physicians, experts and researchers in public health and across the care continuum, this Road Map reflects input from over 200 community members and professionals working in AI/AN communities. This feedback resulted in a focus on health equity through a strength-based approach, the indigenous determinants of health, inclusive imagery and graphics, and expanded examples of public health action from different AI/AN communities.	8,636 hard copies 828 digital downloads

### Engaging public health with the HBI Road Map Series

The HBI engages health departments and tribal health organizations to implement the HBI Road Map Series actions through direct engagement programming and technical assistance. The early years of the HBI focused on strengthening relationships with key staff from health departments and tribal health organizations (Olivari et al., 2020) to make the case for public health action on brain health. As more public health practitioners recognized their role in addressing dementia, the HBI developed capacity-building programming to offer training resources and funding assistance. Today, these engagement programs help to grow public health leaders and support the integration of brain health, dementia, and caregiving issues into health departments and tribal organizations, with influence on their partnerships, resources, organizational priorities, knowledge, and skills (Table 2).

**Table 2.** Alzheimer’s Association Road Map Series engagement programs.

Name	Eligibility	Program goal	Structure	Reach
*Grants (2014–2018), Learning Communities (2018–2019) & Planning Lab (2019–2020)*	State Health Departments	Provide state health agencies with technical and peer support to develop HBI action plans	Varied	4 cohorts, 24 state health departments total
*LHD Learning Collaboration (2019–2020)*	Local Health Departments (LHDs)	Educate health department staff and support development of a local plan to implement HBI actions	12 month cohort of 2 local health departments	1 cohort, 2 local health departments
*HBI Road Map Strategists Program (2022–2025)*	Local Health Departments (LHDs)	Increase the capacity of LHDs to address brain health and dementia Strategists serve as systems change agents to advance population health approaches to dementia.	9-month cohort of up to 10 LHDs LHDs receive $48,000 Program offers education, peer support, and technical assistance Strategists assess their community’s needs and strengths, develop an implementation plan aligned to the HBI Road Map, and train their LHD colleagues about dementia and caregiving	4 cohorts, 36 total Strategists
*HBI Road Map Champions Program (2025)*	Tribally-led health programs and urban Indian organizations	Increase the capacity of public health professionals to address brain health and dementia throughout their communities Advance community-focused population health approaches related to dementia	9-month cohort of up to 15 Champions Champions are public health workers in American Indian and Alaska Native (AI/AN) communities Champions assess their community’s strengths and needs and develop an implementation plan aligned to the HBI Road Map for AI/AN Peoples Programs/Organizations receive $50,000 Program offers education, peer support, and technical assistance	1 cohort, 15 total Champions

As the HBI Road Map Series has evolved, the ability to measure, assess, and evaluate the impact of these public health actions has increased. The Alzheimer’s Association and state health department staff have implemented actions from the Road Map in all 50 states and D.C. The 2023–2027 HBI Road Map introduced an evaluation tool designed to evaluate implementation of the HBI Road Map actions in a coordinated, systematic way. The evaluation tool is centered around the nine outcomes of the HBI Road Map Series. These outcomes provide goals for health departments to improve population health outcomes related to brain health while the evaluation tool provides specific measures for each HBI Road Map action. The standardized nature of the measures allow health departments to track and evaluate impact over time. The nine outcomes are:

1. Increase community partnerships
2. Increase integration with other chronic disease efforts
3. Increase policy action and implementation
4. Increase data availability, quality and utilization
5. Increase data-informed decision making and action
6. Reduce stigma and bias about cognitive decline
7. Increase knowledge and skills of current and future workforce
8. Increase public knowledge about brain health, risk factors for dementia and benefits of early detection and diagnosis
9. Increase public knowledge and use of services for people living with dementia and their caregivers

**Figure 1.**
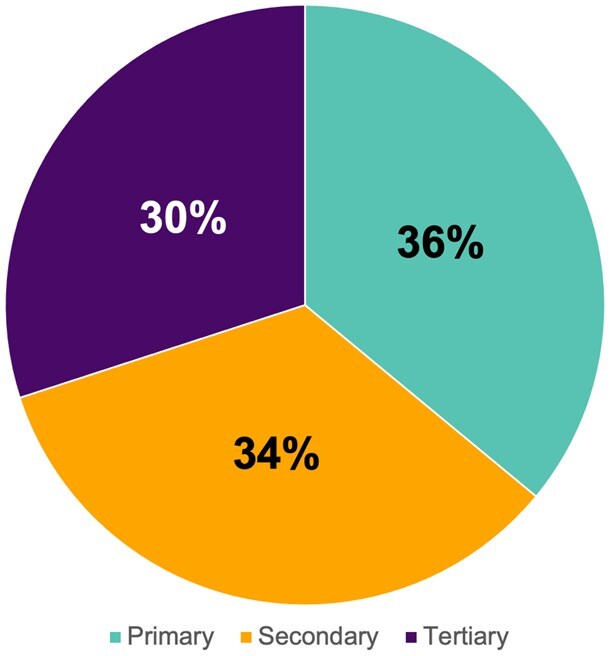
Percentage of total HBI Road Map actions implemented in 2024 among 32 health departments, by prevention level (Alzheimer’s Association, 2025b).

Association staff worked closely with health departments to collect the first and second rounds of Road Map evaluation data in spring and fall 2024. In total, 32 health departments completed the evaluation tool. These health departments are collectively undertaking activities in all four domains and at each level of prevention (Figure 1). Of the participating health departments, 91% have implemented HBI Road Map actions to strengthen partnerships and policies, 100% have implemented actions to measure, evaluate and use data, 75% have implemented actions to build a diverse and skilled workforce, and 88% have worked to engage and educate the public. Looking cumulatively at outcomes measures, 999 community partners were involved in jurisdictional coalitions, 2,956 primary care providers were reached with brain health information; 2,216 public health professionals were trained on brain health and dementia; and 22,422 people received educational programming on brain health.

## Advancing the collection, analysis, and use of public health data

Assessing and monitoring population health is a core function of governmental public health. Through data collection, needs assessment, and analysis, public health officials and policymakers can better understand the impact of Alzheimer’s and dementia and drive positive changes in response. In partnership with the CDC, each state health department annually conducts the Behavioral Risk Factor Surveillance System (BRFSS) survey, a primary source of health information. State health departments have the option to collect data on the prevalence and impact of cognitive decline and caregiving through two supplementary modules that can be added to the core survey:

- **Cognitive Decline Module,** which collects information on self-reported difficulties in thinking or memory–reported as subjective cognitive decline (SCD)—and its effects on function and daily living; and the
- **Caregiver Module,** which collects information about people who provide regular care or assistance to a friend or family member due to disability, disease, or health condition, the types of care provided, and insight into the scope of caregiving responsibilities.

### BRFSS module development and revision

The CDC and the Alzheimer’s Association led the process to develop and pilot versions of the Cognitive Decline and Caregiver Modules in 2009, with nationwide availability of the Cognitive Decline Module beginning in 2011 (Olivari et al., 2021) and the Caregiver Module in 2015 (CDC, 2024b). Since the pilots, the ­Alzheimer’s Association and CDC routinely review and propose revisions to modules to ensure they remain current, the data collected are actionable, and to encourage the modules’ uptake by state health agencies.

Most recently, the Alzheimer’s Association and CDC co-chaired expert workgroups in 2021 and 2022 to review and revise the Cognitive Decline Module and the Caregiver Module, respectively. Workgroups were composed of academic researchers, chronic disease directors, epidemiologists, survey methodologists, policy analysts, and BRFSS coordinators. Each workgroup reviewed the latest scientific evidence on SCD or caregiving, examined data from prior years, discussed the use of the resulting data on state agency programming, and aligned the wording of the modules with current surveillance methodology best practices. The workgroups developed proposed revisions that were then submitted to the CDC Population Health Surveillance Branch for review before being presented to and voted upon by the BRFSS Coordinators for final adoption. Revisions from both workgroups were accepted, and the latest revised modules are available for use in BRFSS surveys beginning in 2023 (Cognitive Decline Module) and 2024 (Caregiver Module).

The Alzheimer’s Association developed a rotating, multi-year campaign to facilitate the cost-effective implementation of the Cognitive Decline and Caregiver Modules and allow for national data analysis. These campaigns focus on one module over a 2-year time period with the goal of all 50 states, the District of Columbia, and Puerto Rico implementing that module at least once during the 2-year campaign. Running since 2011, these campaigns have resulted in consistent implementation of these two modules and allowed data to be analyzed as a nationwide dataset. Over time, states have recognized the value of the data collected, leading to 49 states, the District of Columbia, and Puerto Rico participating in the two most current campaigns. Further, some states have elected to implement either or both modules more frequently and outside of the coordinated campaigns. The data are used annually in state fact sheets and national reports, with 10,490 total downloads of state-based fact sheets since 2010.

### Engaging public health with data

The increasing availability of relevant BRFSS data in state health departments allows states to play an active role in dementia surveillance. In Georgia, BRFSS data were featured in a press conference by a state senator discussing the need for robust public health attention to Alzheimer’s. The BRFSS data informed the development of the Georgia state Alzheimer’s plan, helped establish the need for a state dementia registry, and helped forge a stronger partnership between the state health department and the state aging agency (Georgia State Senate, 2018). In Connecticut, BRFSS data were submitted as testimony to the Aging Committee of the Connecticut General Assembly, highlighting the need for expanded respite care services in the state. The BRFSS data highlighted the difficulties dementia caregivers face when performing caregiving responsibilities (Testimony of Christy Kovel, Director of Public Policy, 2022). In conjunction with BRFSS data, health departments have opportunities to expand the use of other state datasets, such as claims data and hospitalization data, to further explore the impact of dementia in communities.

### Healthy People 2030

Healthy People 2020 was the first to include dementia objectives. These objectives focused on increasing disclosure of a dementia diagnosis among people living with dementia or their caregivers, and reducing preventable hospitalizations among people living with dementia (Office of Disease Prevention and Health Promotion, n.d.a). A third dementia objective was added to Healthy People 2030 (HP2030) focused on increasing conversations among people with SCD and their health care provider (Office of Disease Prevention and Health Promotion, n.d.b). The current dementia-specific objectives for HP2030 are:

1. **DIA-01:** Increase the proportion of older adults with dementia, or their caregivers, who know they have it.
2. **DIA-02:** Reduce the proportion of preventable hospitalizations in older adults with dementia.
3. **DIA-03:** Increase the proportion of adults with SCD who have discussed their symptoms with a provider.

The Alzheimer’s Association analyzed baseline and midpoint measurements for DIA-01 and DIA-02 with an external data analytics firm and is supporting the analysis of DIA-03. This forthcoming analysis uses a new methodology allowing public health agencies to examine diagnosis disclosure rates and preventable hospitalizations among people living with dementia by a variety of demographic characteristics and on a state-by-state basis.

## Training the current and future public health workforce

A skilled, diverse, and representative workforce is essential to building public health capacity and improving population health outcomes. Historically, public health and health care professionals have received little or no formal training on dementia during their education (Olivari et al., 2020). Likewise, shifting demographics toward an older population have not been met with a response in academic institutions, with few offering training in aging or gerontology (Kaskie & Lepore, 2023). In response, the Alzheimer’s Association led the development of *A Public Health Approach to Dementia–*a free public health curriculum on dementia. The curriculum’s modules introduce students from public health and allied disciplines to topics central to the HBI including health equity, risk reduction, early detection, and caregiving as well as provide introductory modules on the public health approach to dementia and the HBI Road Map Series. The curriculum features implementation strategies, public health professionals sharing their perspectives, and the voices of people with lived experience in caregiving and cognitive decline.

Originally developed in 2016, the curriculum was released as static slide presentations before evolving to include recorded video presentations, worksheets, and discussion guides. In 2023, the curriculum was revised and migrated to an online, interactive learning environment to promote accessibility, learner engagement, and evaluation. Each online module includes videos and knowledge checks, and is accompanied by an instructor guide for faculty educators to incorporate content from the module into their classroom or teaching environment. Instructor guides include test questions, video resources, and additional learning activities designed to increase classroom discussion on dementia. Upon module completion, learners receive a certificate of completion and can receive free Certified Health Education Specialist (CHES) continuing education credits through the American Public Health Association.

### Educating the current and future public health workforce

The reach of the curriculum has expanded substantially over time. Between 2016 and 2024, the first iteration of the curriculum had 655 total downloads and 34,807 total YouTube views. Launched in fall 2023, the online iteration of the curriculum has reached over 2,400 individuals–including students, health department personnel, and non-profit staff. Increasingly, dementia-specific jurisdictional coalitions are using the curriculum to train their membership on the public health approach to dementia. Results from the post-module evaluations show 92% of learners report their knowledge has increased and 89% report they are likely to apply what they have learned in their work or studies in the near future (Table 3).

**Table 3.** Reach and impact of select Alzheimer’s Association HBI communications.

Alzheimer’s Association communication activities, 2020–2024	Reach	Impact
13 webinars	3,163 attended	Advanced education among the public health community and connected them with resources on a range of topics, including brain health, dementia, caregiving, public health data, emergency response, Healthy People 2030, health improvement planning, and other public health topics. Among participants, 94% of attendees agreed or strongly agreed that the webinar increased their knowledge of the topic. 93% agreed or strongly agreed that the webinar was valuable to their work.
64 conference presentations Examples of conferences with HBI information presented include annual meetings of: American Public Health Association, Gerontological Society of America, Society for Public Health Educators, BRFSS Coordinators, National Association of County and City Health Officials, Title VI Conference, and the National Indian Council on Aging	1,798 individuals attended presentations	Grew awareness of the HBI and topics across spectrum of professional disciplines including: public health, aging, public health education, health department practitioners, data and implementation science, tribal researchers
Monthly Alzheimer’s Public Health Newsletter	Average 6,677 monthly subscribers	Across 50 editions, 45.73% open rate.
Public Health Curriculum	2,909 enrollments across 4 modules	92% of learners who completed a post-module survey reported that their knowledge on the topic was higher or much higher, and 89% of learners reported that they would be able to use what they learned in the module in their work, classes, or studies.

## Impact of the Healthy Brain Initiative

After the establishment of the HBI in 2005, additional federal and state policies have continued to shape the public health approach to Alzheimer’s and dementia. In 2011, the National Alzheimer’s Project Act was signed into law, leading in 2012 to publication of the first *National Plan to Address Alzheimer’s Disease* and a path toward a coordinated, federal response to dementia (National Alzheimer’s Project Act, Public Law 111—375, 2011). In 2021, the National Plan expanded to include a new sixth national goal focused on dementia risk reduction (US Department of Health and Human Services, 2021). These efforts have continued to the present day, with the NAPA Reauthorization Act signed into law in 2024 (NAPA Reauthorization Action, Public Law 118—92, 2024).

In 2018, Congress passed the Building Our Largest Dementia Infrastructure for Alzheimer’s Act (BOLD Infrastructure for Alzheimer’s Act, Public Law 115—406, 2018). Implemented by the CDC, the BOLD Act utilizes the HBI framework and strategy to expand public health capacity by establishing expert-led Public Health Centers of Excellence on dementia risk reduction, early detection, and caregiving; providing funding to health departments of states, political subdivisions of states, and tribes and tribal organizations; and increasing the timely collection and reporting of public health data. The passage of the BOLD Reauthorization Act of 2024 (BOLD ­Reauthorization Act, Public Law No. 118—142, 2024) is further evidence of sustained commitment to a public health approach to dementia at a national level.

Policy change has also evolved at a state level, with state governments increasingly prioritizing dementia in legislation and planning. Every state is currently enacting its own ­Alzheimer’s strategic plan. Concurrently, a growing number of state governments are allocating state funds toward dementia initiatives, demonstrating greater attention and focus on dementia as a public health issue. In 2023, 39 states and the District of Columbia appropriated a total of $247.9 million state dollars to dementia-specific initiatives in states (Alzheimer’s Association, 2024). These policy achievements relied, in part, on major public health advances supported by coordinated action through the HBI—and their passage has accelerated the public health response to Alzheimer’s and dementia for the residents of respective jurisdictions.

## Conclusion

In 20 years, the HBI has laid the foundation for addressing Alzheimer’s as a public health issue by creating a framework for health departments and tribal health organizations to improve population outcomes related to brain health, dementia, and caregiving throughout their communities. Over the next 20 years the population will continue to grow older, and scientific advancements will continue offering more successful early detection measures, treatments, and continue the hope for the first Alzheimer’s survivor. As scientific advancements continue, public health approaches will be critical to ensure access to early detection, treatment, support and healthy living are available in all communities. Successful medical advancements cannot impact population health without public health. The HBI will continue to use data, train the workforce, and evolve the framework of public health action to ensure health departments, tribal health organizations, and national partners are able to provide their communities with the opportunity to live life with the healthiest brain possible.

## Data Availability

This article does not report data and, therefore, the pre-registration and data availability requirements are not applicable.
